# Near-Peer Tutor: A Solution For Quality Medical Education in Faculty Constraint Setting

**DOI:** 10.7759/cureus.16416

**Published:** 2021-07-16

**Authors:** Meenakshi Khapre, Rupinder Deol, Anusha Sharma, Dinesh Badyal

**Affiliations:** 1 Social Preventive Medicine, All India Institute of Medical Sciences, Rishikesh, IND; 2 College of Nursing, All India Institute of Medical Sciences, Rishikesh, IND; 3 Community and Family Medicine, All India Institute of Medical Sciences, Rishikesh, IND; 4 Pharmacology, Christian Medical College, Ludhiana, IND

**Keywords:** peer group, peer learning, health science students, knowledge, skill, satisfaction, systematic review, meta-analysis

## Abstract

Near-peer mentoring is a formal relationship in which more qualified students guide immediate junior students. It is an innovative approach to increase students' engagement from varied backgrounds and cultures in the health profession. This systematic review and meta-analysis aimed to investigate and compare the effectiveness of near-peer tutoring and faculty/expert teaching in health science undergraduates on knowledge and skill outcome. The review question considered was "how effective is near-peer tutor compare to faculty/expert teaching for undergraduate health science students?" A comprehensive systematic search was undertaken in PubMed, Embase, Scopus, and Cochrane and screened initially in Rayyan software (Qatar Computing Research Institute, Qatar). Identified articles were screened independently for eligibility by two reviewers and extracted the data. Data were analyzed using standardized mean difference with Review manager version 5.5 (Cochrane Campbell Collaboration).

Sixteen studies were analyzed. Heterogeneity (I^2^) among studies was high in knowledge and skill scores. Heterogeneity was reduced by 30-40% after sensitivity analysis. No difference in knowledge and skill score was found among the near-peer and expert teaching groups. Students had a satisfactory learning experience with near-peer tutors except for some issues related to teaching proficiency in near-peers. Near-peer teaching was found to be as effective as faculty/expert teaching. Students were more comfortable with near-peers. As mentioned by students, some challenges were differences in teaching skills and level of knowledge among near-peers.

## Introduction and background

Near-peer teaching is becoming increasingly popular in medical education [1,2]. It is a voluntary collaboration between colleagues of almost similar rank, one or more years senior to another, and common academic interests. The immediate senior may facilitate discussions, provide personal support and feedback, while the senior clinician/faculty may oversee the mentoring process [1]. Near-peer teaching is a formal relationship in which a more qualified student provides guidance and support to another student [2]. 

Near-peer teaching is an innovative approach to increase students' engagement from varied backgrounds and cultures in the health profession field, addressing the lack of diversity in healthcare and the shortage of teaching faculty [1]. Effective near-peer mentoring nurtures long-term professional friendships and professional collaborations between peers [3]. We echo with an author that being close to the new learner's social, professional, age level, the near-peer mentor may influence the cognitive and psychomotor learning of the new learner in a better manner [2]. Also, the new learners may feel more comfortable asking queries and talking to someone with a lesser age gap. 

Near-peer is a cost-effective alternative to expert teaching as healthcare fraternities are facing a shortage of faculty tutors and mentors. Various medical and nursing councils like General Medical Council (UK), American Medical Association (US), and National Medical Commission (India) emphasize that the medically trained personnel should also be effective teachers and communicators. Near-peer tutoring may be more beneficial for the new medical entrants as new students would be less hesitant to share their difficulties with near-peers than with faculty [3]. Notwithstanding these apparent benefits, near-peer tutoring is time-consuming. It places additional demands and responsibilities on near-peer tutors, including leadership, prioritization, and identifying and coping with their own mistakes or weaknesses. This is especially important to consider when there is high stress among medical students due to heavy load of studies [4].

Many studies related to near-peer teaching are available, but a limited sample size in them may be a reason for inability to obtain a significant difference in learning outcomes among near-peer and expert groups. A systematic review by Rees et al. compared peer teaching to faculty teaching, where peers can be from the same or higher academic years [5]. We included only those peers who are at a higher academic level than study participants, i.e., near-peer tutors. We consider that near-peer tutors have gone through the same learning experiences as study participants in the recent past and therefore are in a better position to guide undergraduate students. 

Objectives

The present study aimed to review the studies done on the effectiveness of near-peer mentoring and compare the effectiveness of senior near-peer tutoring and faculty (expert) teaching in health science graduates in terms of knowledge and skill score. We performed the narrative synthesis on the perceived satisfaction of students due to near-peer tutoring. 

Research question

The review question considered was "how effective is a near-peer tutor compared to faculty/expert teaching for undergraduate health science students regarding improvement in knowledge, skill, and satisfaction?" Other questions that we tried to address by this review were (1) what are the reported benefits of near-peer tutoring? (2) What are the reported challenges in utilizing near-peer tutoring as a formal teaching-learning (T-L) strategy in medical education. 

## Review

We report this systematic review following guidelines of the Preferred Reporting Items for Systematic Reviews and Meta-Analyses (PRISMA) checklist (Appendix) [6]. We conducted a systematic review from March 2020 to March 2021. 

Eligibility criteria**

This review encompassed a wide range of experimental and quasi-experimental study designs, but not limited to randomized controlled trials, non-randomized controlled trials, pre-post studies, and comparative observational studies. Previous meta-analyses/reviews, editorial comments, and opinion pieces were excluded. Participants included were undergraduate health professionals from medicine, dental, nursing, and physiotherapy courses. Studies that included post-graduate students were excluded. We included studies where teaching/learning session conducted by senior/junior peer tutor as given by Bulte et al. and faculty/expert were compared for an outcome like knowledge or skill with/without student or peer satisfaction [7]. We defined peer tutor as one or more years senior to the trainee on the same level of health education training comprising junior residents (senior near-peer) and students (junior near-peer). Near-peer tutors can be interns, residents, demonstrators, tutors of senior-level using any method of instruction. Studies related to peer tutoring by same level, reciprocal peer tutoring, or class-wide peer tutoring were excluded from this review. We did not exclude the studies based on methodological quality because we planned to conduct sensitivity analysis by removing low-quality studies. We excluded studies where the full articles could not be retrieved despite our best effort and studies published in a language other than English. 

Search strategy

We performed a comprehensive search to identify potentially relevant published research studies. PubMed, Embase, Scopus, and Cochrane were searched for studies published until March 2021. The search strategy was defined through the principles of a systematic search, using the population, intervention, comparison, outcome (PICO) scheme. The search term included the following keywords, i.e. "peer tutor*" "peer learn*" OR "peer teach*" AND "medicine OR medical OR nursing OR 'health science' OR dental". We could not find the medical subject headings (MeSH) term for near-peer. A detailed search strategy is given in Table 1. 

**Table 1:** Search strategy

Search engine/database	Search strategy
PubMed	"Peer teaching"[All Fields] OR "peer learning"[All Fields] OR "peer assisted"[All Fields] OR “peer tutoring” AND (Clinical Trial [ptyp] OR Controlled Clinical Trial [ptyp] OR Comparative Study[ptyp])
Embase	#4 AND (“clinical trial”/de OR “comparative effectiveness”/de OR “comparative study”/de OR “control group”/de OR “controlled clinical trial”/de OR “controlled study”/de OR “crossover procedure”/de OR “feasibility study”/de OR “human experiment”/de OR “major clinical study”/de OR “pilot study”/de OR “pretest posttest design”/de OR “randomized controlled trial”/de)peer2020-01-172020-01-16112 #4(“peer tutor*”:ab,ti OR “peer learn*”:ab,ti OR “peer teach*”:ab,ti OR “near pear”:ab,ti) AND (medicine:ab,ti OR medical:ab,ti OR nursing:ab,ti OR “health science”:ab,ti OR dental;ab,ti) AND (faculty:ab,ti OR expert*:ab,ti OR clinician:ab,ti OR senior:ab,ti OR junior:ab,ti OR resident:ab,ti)peer2020-01-172020-01-16341 #3#2 AND (“comparative effectiveness”/de OR “comparative study”/de OR “meta analysis (topic)”/de OR “pilot study”/de OR “randomized controlled trial”/de OR “systematic review”/de)peer2020-01-112020-01-11127 #2”peer teaching”:kw OR “near peer”:kw OR “peer review”:kwpeer2020-01-112020-01-111320
Cochrane	"peer tutor*" OR "peer learn*" OR "peer teach*" in Title Abstract Keyword AND medicine OR medical OR nursing OR “health science” OR dental in Title Abstract Keyword AND faculty OR expert OR clinician OR senior OR junior OR resident OR doctor in Title Abstract Keyword - (Word variations have been searched)
Scopus	TITLE-ABS-KEY ("peer learn*" OR "peer teach*" OR "peer tutor*") AND TITLE-ABS-KEY (medical OR medicine OR nursing OR "health science" OR dental) AND TITLE-ABS-KEY (expert OR faculty OR resident OR senior OR junior OR doctor OR tutor OR demonstrator) AND NOT INDEX (medline) AND (LIMIT-TO (DOCTYPE, "ar")) AND (LIMIT-TO (SUBJAREA, "MEDI") OR LIMIT-TO (SUBJAREA, "NURS") OR LIMIT-TO (SUBJAREA, "HEAL")) AND (LIMIT-TO (LANGUAGE, "English"))

Study selection 

All searched result citations were loaded into and managed in Zotero bibliography software (Center for History and New Media, George Mason University, Fairfax County, VA) and duplicated articles were removed. Then it was being uploaded to Ryaan software for initial screening. After gathering the evidence, identified references were screened independently for eligibility by two reviewers using a three-stage approach with title, abstract, and full text. No stipulation was made regarding the duration or frequency of the educational program. Any type and mode of teaching-learning method were included. We resolved discrepancies during the selection process by discussion. We recorded and reported, reasons for excluding studies following the full-text review. References of included studies were hand-searched to identify any further relevant references. 

Data collection process**

Two trained investigators in systematic review (MK and RD) completed data extraction independently using the validated data extraction form and later compared the consensus data. Data extraction form contained information on the author, year, place of study, study design, participants characteristics (year of education and stream of health sciences), intervention (year of residency or medical education, volunteered/not volunteered), comparator group (faculty/expert clinician), teaching-learning method used, knowledge and skill pre and post-test score in both the groups (mean and SD), satisfaction score (mean and SD if available), benefits perceived and challenges encountered. Any disagreements that arose between the reviewers were arbitrated by consensus.

If the data in a study of last five years (since January 1, 2014) were found to be unclear, missing, or presented in a non-extractable or unusable form, then we contacted the authors of studies for clarification via email and followed up after two weeks in case of no response. The authors of studies prior to January 2014 were not contacted. 

Data items 

Articles with variables knowledge, skill, satisfaction measured quantitatively or qualitatively were included. Benefits and challenges as perceived by students and peers were noted along with recommendations suggested by the author.

Quality assessment of studies 

We assessed the methodological quality of the study using the Medical Education Research Study Quality Instrument (MERSQI) [8]. MERSQI and Newcastle Ottawa scale-education (NOS-E) are useful, reliable, complementary tools for appraising the methodological quality of medical education research [9]. The perfect MERSQI score was 18. We considered more than 10.5 MERSQI scores as an acceptable quality of the study [10].

Data synthesis 

We performed the narrative synthesis with a summary of included studies as a preliminary step. We then tabulated the results to identify the pattern and explain the differences in results between studies. We plotted the effect estimate in forest plot considering the standardized mean difference of knowledge and skill post-test score between expert and near-peer groups. We confirmed that the pre-test score was not significantly different in both the groups, either as reported by the author or calculated t-test or Z-score. If a significant difference was found at a baseline test score, a changed score was entered for meta-analysis. We tested the degree of heterogeneity with Cochrane Q and I^2 ^statistics. We planned a sensitivity analysis considering the methodological quality if statistical heterogeneity was more than 50%. We considered plotting funnel plot for publication bias only when we can include more than 10 studies in meta-analysis. The results were statistically significant when two-sided p-values were less than 5%. All analyses were conducted in Review manager version 5.5 (Cochrane Campbell Collaboration). 

Results

Selection of Sources of Evidence

**Figure 1:**
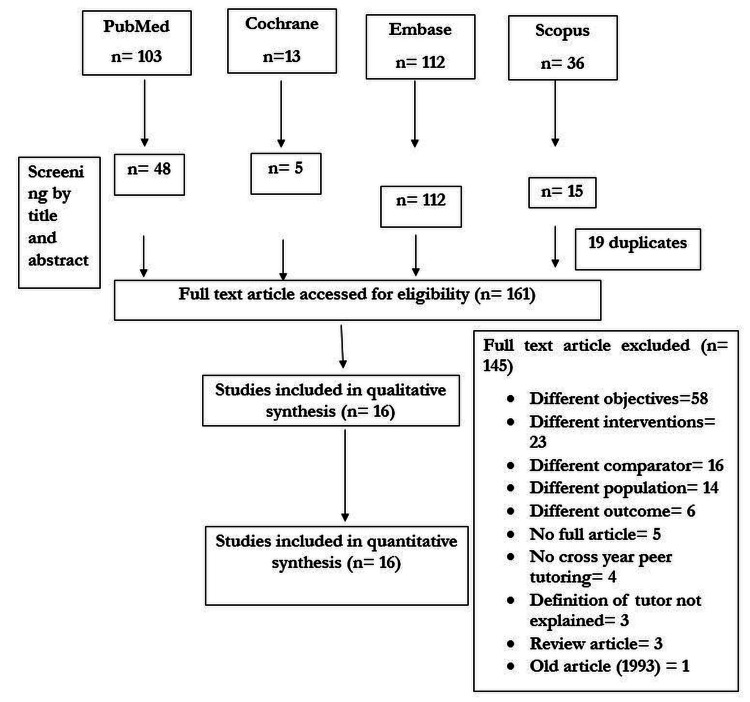
PRISMA flow chart of study selection process PRISMA: Preferred Reporting Items for Systematic Reviews and Meta-Analyses

Based on the primary search from selected databases, a total of 264 studies were identified. After removing duplicates (n=19) and screening by title and abstract to remove review articles, letter to the editor, or study done on students other than health graduates, a total of 161 were eligible for full-text review. Selected articles were assessed for eligibility criteria and 145 were excluded. Among excluded studies, 58 studies had objective other than effectiveness of near-peer tutor; in 23 studies, the intervention was not peer group; in 16 studies, the comparator group does not consist of expert/faculty; and in 14 studies, the study population was other than health stream students. The meaning of peer tutor was not clear in three studies; three were review articles. The meaning of peer tutor was not clear in three studies; three were review articles. Finally, we identified 16 studies for systematic review and meta-analysis (Figure 1).

Characteristics of Sources of Evidence 

The characteristics of all included studies are summarized in Table 2. Medical sciences students were the study population in all included studies except three where one study included dental students [11], one nursing [12], and one sports medicine (physiotherapy) [13]. All included studies were randomized experimental designs except one study [14]. In the study by Cameroon et al., though mentioned cluster-randomized trial, the unit of study was an individual student [11]. In the study by Beck et al., historical control of the previous year was enrolled [15]. As per inclusion criteria, the near-peer tutor was defined as one or more years senior to the trainee on the same level of health education training comprising junior residents (senior near-peer) and students (junior near-peer). In seven studies, the near-peer were volunteers [11,14,16-18]. Only in research done by Heckmann et al., peers were trained [19]. All the studies compared teaching by experts and faculty members except in a study done by Graziano, where senior resident (Master of Surgery) was in the comparator group [20]. The teaching-learning method was the same in both study groups except in Beck et al., where the intervention group received small-group teaching and the comparator group received large group teaching [15]. Teaching was conducted via a simulation laboratory in Adam et al. [14] and Graziano [20] and a laboratory setting in Weidner and Popp [13] and Weyrich et al. [21]. In a study done by Adam et al. and Heckmann et al., students' skill and satisfaction were assessed, while in other studies, either knowledge and skill were assessed with or without satisfaction [14,19]. 

**Table 2:** Characteristics of included studies T-L: teaching-learning; OSCE: objective structured clinical examination; R: reported; BDS: Bachelor of Dental Surgery; PBL: problem-based learning

S no.	Author, year	Subject	Population	Type of study	T-L session delivered by (interventional group)	T-L session delivered by (comparator group)	T-L method	Outcome measure	Knowledge	Skill	Satisfact-ion
1	Adam et al. 2018 [14]	Internal medicine	Interns	Non-randomized interventional	Resident instructor	Faculty	Simulation	Debriefing Assessment for Simulation in Healthcare (DASH Survey), pre and post knowledge test	R	R	R
2	Beck et al., 2016 [15]	Histopathology	M1 first-year medical student (2010-2014)	Experimental historical control previous year	M2 second-year medical student	Faculty	Interventional: small group modular Comparator: large group lecture	Quiz: 25% of questions relating to factual information and 75% of questions related to application, satisfaction of students	R		R
3	Ben-Sasson et al., 2019 [22]	Cardiac ultrasound	Internal medicine rotation for 3 months	Prospective interventional study- randomized	Third-year of clinical education	Board-certified cardiologist and a diagnostic medical sonographer	Small group	Six-minute practical exam to assess their image-acquisition skills		R	
4	Blank et al., 2013 [23]	Physical examination	Third-year medical student	Randomized trial	Fourth-or fifth-year volunteered students	Physician lecture plus web-based tutorial	Tutorial	OSCE after 2 months		R	
5	Brannagan et al., 2013 [12]	Clinical lab environment	First-year nursing students	Randomized trial	Senior-level nursing students with faculty instruction	Faculty only	Lecture, laboratories	Cognitive evaluation pre-and post-tests	R		
6	Buscher et al., 2013 [24]	Newborn examination	Medical students	Randomized trial	Senior peer	Senior lecturer	Small group	Modified OSCE		R	R
7	Cameron et al., 2015 [25]	Dental Task 1: clinical task of impression Task 2: preclinical handpiece skills	BDS first-year	Cluster randomized controlled trial design (unit of study was individual)	Volunteered BDS –fifth-year	Clinical teaching staff	Small group	OSCE after 1 week		R	R
8	Graziano, 2011 [20]	OBGY operating room introduction course	Third-year	Randomized trial	Fourth-year	Resident Assisted learning	Simulation training	Objective exercises		R	
9	Heckmann et al., 2008 [19]	Clinical skills training during neurology clerkship	Medical student	Randomized trial	Senior peer trained	Postgraduates-trained group	Small group	Written test and objective structured clinical examination (OSCE) 2. Self-estimated gain in competence	R	R	R
10	Hudson and Tonkin, 2008 [26]	Clinical skills program	Second-year medical student	Randomized controlled trial in 3 teaching hospitals	Sixth-year medical student	General medical practitioner	Not mentioned	End-of-year summative assessment of clinical skills		R	
11	Kemper et al., 2014 [27]	Mirror course for 6 hours in 5 days otorhinolaryngology (ORL)	Medical students (fifth year)	Interventional Randomization: not reported	Final-year student	Physician	Not mentioned	OSCE		R	R
12	Matthes et al., 2002 [16]	Pharmacology	Medical student (third-year)	Cluster randomized controlled trial design	Volunteered medical students (fourth-year or higher)	Expert faculty	PBL	Written examination consisted of 20 multiple-choice questions and 10 short-essay questions	R		R
13	Nomura et al., 2017 [17]	Communication training	Fourth-year medical students	Mixed method randomized controlled non-inferiority trial	Fifth-year students, voluntary with no financial incentives	Faculty	Skill-based small group teaching	OSCE was conducted one week after the training session		R	
14	Weidner and Popp, 2007 [13]	Upper extremity injury evaluation athletic training education program	Undergraduate students who were enrolled over the spring 2004 and spring 2005 semesters	Randomized, pre-and post-test experimental design	Upper-division athletic training majors	Approved clinical instructor	Laboratory course instruction and review sessions for two weeks	Six psychomotor skill Athletic Training Peer-Assisted Learning Assessment Survey		R	R
15	Weyrich et al., 2009 [21]	Training in technical procedures	Volunteer third-year medical students	Randomized trial	Fourth-year tutor	Faculty	Skill laboratories	OSCE binary checklist		R	
16	Widyahening et al., 2019 [18]	Critical appraisal skills learning program	Fourth-year medical students	Randomized crossover trial	Voluntary near-peer tutors	Experienced medical staff	Tutorials	Evidence-based practice confidence scale, written test, attitude	R		R

Quality Assessment of Study 

Table 3 shows the study quality assessed with MERSQ1. The score ranged from 10.5 to 14.5, with a mean of 12.5 (1.095) and a median of 12.5. None of the studies scored below the predetermined cut-off level of 10.5%. A study carried out in three teaching hospitals scored highest for methodological quality [26]. A study done by Blank had a response rate of less than 50% [23]. Five studies scored zero under the heading validity of the evaluation instrument as studies did not mention the statement on the content of instrument and validity. None of the included studies studied long-term outcomes in terms of patient benefit.

**Table 3:** Study quality assessed with MERSQI MERSQI: Medical Education Research Quality Instrument

Sr no	Author, year	Study design	Sampling	Type of data	Validity of evaluation instrument	Data analysis	Outcome	Total
1	Adam et al., 2018 [14]	2	2	3	1	3	1.5	12.5
2	Beck et al., 2016 [15]	2	2	3	1	3	1.5	12.5
3	Ben-Sasson et al., 2019 [22]	3	1	3	0	2	1.5	10.5
4	Blank et al., 2013 [23]	3	1 (response rate <50%)	3	1	3	1.5	12.5
5	Brannagan et al., 2013 [12]	2	1	3	1	3	1.5	11.5
6	Buscher et al., 2013 [24]	3	2	3	0	3	1.5	12.5
7	Cameron et al., 2015 [25]	3	2	3	1	3	1.5	13.5
8	Graziano, 2011 [20]	3	2.5	3	2	2	1.5	14
9	Heckmann et al., 2008 [19]	3	1	3	0	2	1.5	10.5
10	Hudson and Tonkin, 2008 [26]	3	3	3	1	3	1.5	14.5
11	Kemper et al., 2014 [27]	2	2	3	1	3	1.5	12.5
12	Matthes et al., 2002 [16]	3	2	3	0	3	1.5	12.5
13	Nomura et al., 2017 [17]	3	2	3	0	3	1.5	12.5
14	Weidner and Popp, 2007 [13]	3	2	3	1	3	1.5	13.5
15	Weyrich et al., 2009 [21]	3	2	3	1	3	1.5	13.5
16	Widyahening et al., 2019 [18]	3	2	3	2	3	1.5	14.5
		Mean (SD)						12.5 (1.095)
		Median						12.5

Results of Individual Sources of Evidence 

Reviewers input post-test scores of all included studies in the current metanalysis as they could not find differences in baseline scores among near-peer and expert groups.

**Figure 2:**
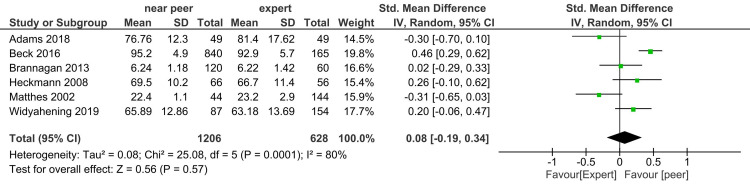
Forest plot of student's knowledge score in near-peer and expert teaching groups

**Figure 3:**
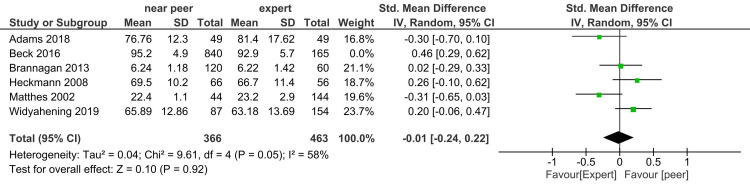
Sensitivity analysis forest plot of student's knowledge score in near-peer and expert teaching groups

Knowledge: Forest plot of the student's knowledge score with six studies (sample size of 1206 in near-peer and 628 in expert teaching group) shows no difference in effect size 0.08 (-0.19, 0.34) (Figure 2). The heterogeneity (I^2^ statistic) was 80% in fixed-effect model that remained unchanged in the random-effect model. Sensitivity analysis after dropping the study done by Beck et al. as MERSQI score was lowest, i.e., 10.5, heterogeneity decreased to 58%, no difference in knowledge score was found (Figure 3).

**Figure 4:**
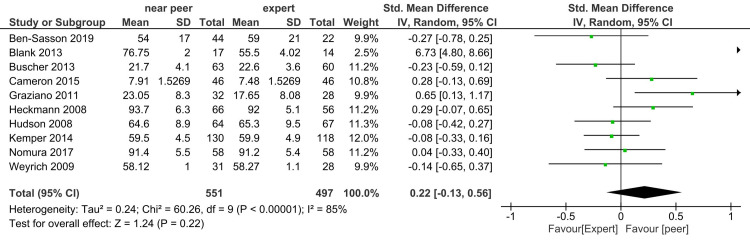
Forest plot of student's skill score in near-peer and expert teaching groups

**Figure 5:**
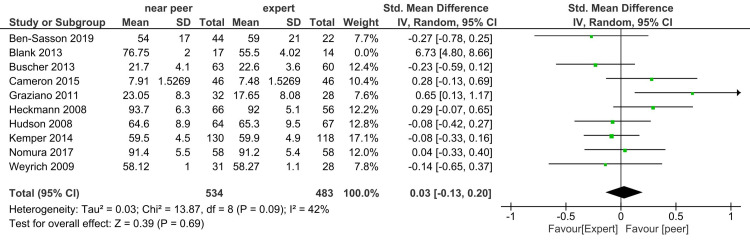
Sensitivity analysis forest plot of student's skill score in near-peer and expert teaching groups

Skill: In Figure 4, when 10 studies were included, the I^2^ statistic was 85% and the estimate was imprecise (-0.13, 0.56). Heterogeneity remained unchanged in the random-effect model. The total sample size was 551 in the intervention group (near-peer) and 497 in the expert group. The study done by Blank et al. favored the near-peer with standardized mean difference (SMD) of 6.73 (4.8-8.66) [23]. We excluded the study done by Blank et al. because the response rate was less than 50%, so the study result might not be reliable. Figure 5 shows no difference in skill score in near-peer and expert teaching groups, I^2^ statistic decreased to 42%. 

Satisfaction: Eight included studies conducted satisfaction surveys among the learners for perceived satisfaction, opinion, experiences, or learning effect. These surveys were in the form of using Debriefing assessment survey or evidence-based practice score, Likert scale or yes/no, agree/disagree dichotomous type questions Kemper found improved perceived learning effect [27]. Other studies showed that a higher proportion of students were satisfied as it was stimulating [20] and less anxious, felt more confident, and enhance collaborative skills [13]. Satisfaction survey scores among the near-peer teaching and expert teaching were found to be non-significant [14,16,18]. 

**Figure 6:**
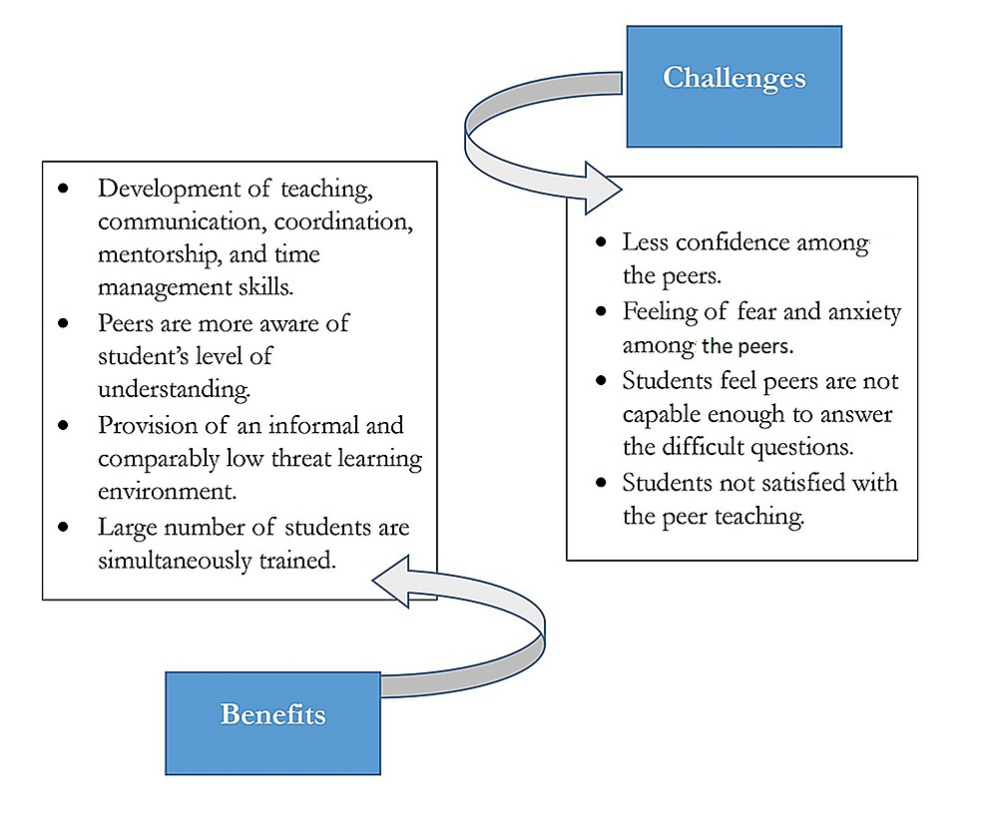
Benefits and challenges of near-peer teaching

Perceived benefits: The benefits are mentioned for peer tutors, learners, and administrators. Peer tutor's benefits were developed teaching, communication, coordination, mentorship, and time management skills. Peer tutors had the opportunity for a deeper understanding of the taught subject. Students felt more comfortable with peers who were approachable and well aware of student level of understanding. The learning environment was of comparably low threat and informal. An overall student gave positive feedback for near-peer teaching. In terms of administration, peer teaching not hindered daily ward rounds, a large number of students were trained simultaneously (Figure 6).

Perceived challenges: The authors had also encountered some challenges like some of the student-expressed dissatisfaction with peer teaching. They felt peers were not trained and not compatible to handle the difficult questions posed by students. Peer tutors themselves felt anxiety and not confident in teaching. In many studies, peers did not teach voluntarily but were forced due to curriculum structure. 

Discussion

Summary of Evidence 

The main finding of the present systematic review was the absence of any significant difference between the near-peer teaching and expert teaching groups in terms of knowledge and skill scores. Studies on comparison of near-peer and expert were existing but with small sample sizes. The present study is the only study to the best of our knowledge where meta-analysis is conducted for the effectiveness of near-peer teaching. Our review's result is in line with a 15-year literature review of peer-led basic life support (BLS) course that demonstrated excellent learning outcomes, cost-effectiveness compared to experts, and peers were more approachable [28]. Near-peer tutoring programs like student grand round, Acute Care Skills Training (ACST) course, and Clinical Skills and Simulation course were found to be effective in enhancing the knowledge and skills of both near-peer and students and were well-received by students [29-31]. 

Even though the methodological quality of included studies was good, we found considerable heterogeneity in knowledge score, which may be due to different settings, programs, populations, and characteristics of near-peers. A study by Beck et al. was done with a historical cohort of the previous four years as control [15]. This time frame may be a source of heterogeneity and when the study was removed from the meta-analysis of knowledge score, it resulted in moderate heterogeneity of 58%. Similarly, for skill scores, the heterogeneity was considerable remained unexplained by the random effect model. The study by Blank et al. was omitted from analysis due to a response rate of less than 50%, which means maybe the only student who was well-prepared may have appeared in the assessment [23]. Though the study mentioned non-response rate was the same in both the study group, the final result may be biased. The forest plot of skill scores on sensitivity analysis also showed no difference in skill scores in both study groups with moderate heterogeneity. The satisfaction of students in our review is in line with Liew et al. that demonstrated consistently higher scores in near-peer evaluation by a large number of students (n=985), suggesting that students benefited from near-peer teaching sessions [31]. 

Volunteered peers participated in two of the included studies [23,25]. The volunteer nature of these programs may have drawn motivated peer tutors and may have a comparable higher score in peer-group than the expert. Wadoodi and Crosby also highlighted that near-peer voluntary recruitment creates an opportunity for participation from potential peers motivated to teach [32]. Near-peers were trained in only one study, which was considered a limitation and challenge in other studies [19]. Training will further improve performance, confidence, decrease anxiety, and role clarity among near-peer tutors. 

Near-peer teaching mutually benefits both tutor and tutee. In our included studies, tutors felt that they could revisit previously learned topics while refining key proficiencies required by different health graduates, such as teaching skills, time management, and leadership. The tutee had also been benefited from advice from previously successful students with first-hand experience in their exams, teaching done at an appropriate level, and a comfortable and safe learning environment. This confirms the hypothesis "cognitive congruence" given by Lockspeiser et al. [33]. However, the authors of included studies encountered various challenges regarding administration (sustainability of program), untrained, incompetent peers. Standardization in the quality of teaching by different peers should be ensured. We further believe that if a near-peer program is initiated as per tips given by Wadoodi and Crosby on tutor selection, training the tutor, and running and evaluating the sessions will further make near-peer a valuable source for medical colleges [30,34]. 

Limitations: Reviewers could not ascertain the reporting bias due to fewer studies. Full article could not be retrieved in five studies, and few studies did not clarify the qualification of peer in comparison to study participants. This review only included the studies in the English language, which means that the studies published in another language were not analyzed. There is always a possibility that the systematic search did not acquire all relevant literature as per inclusion criteria. 

## Conclusions

There was no significant difference in knowledge and skill scores among students taught by near-peers compared to faculty/expert. Overall, students were satisfied with the near-peers teaching skill and their ability to create a comfortable learning environment. As the process of near-peer teaching benefits tutor, tutee, and administrator (due to limited faculty members), peer teaching should be strengthened by orienting students to basic medical education technology in early medical and hand-holding interested students to develop their teaching-learning skills further. Near-peer programs should be part of curriculum delivery right from students entering the health education field to post-graduate trainees.

## Appendices

**Table 4:** PRISMA 2020 checklist PRISMA: Preferred Reporting Items for Systematic Reviews and Meta-Analyses

Section and topic	Item #	Checklist item	Location where item is reported
Title			
Title	1	Identify the report as a systematic review.	Pg 1
Abstract			
Abstract	2	See the PRISMA 2020 for Abstracts checklist.	Pg 2
Introduction			
Rationale	3	Describe the rationale for the review in the context of existing knowledge.	Pg 3
Objectives	4	Provide an explicit statement of the objective(s) or question(s) the review addresses.	Pg 3
Methods			
Eligibility criteria	5	Specify the inclusion and exclusion criteria for the review and how studies were grouped for the syntheses.	Pg 4
Information sources	6	Specify all databases, registers, websites, organizations, reference lists, and other sources searched or consulted to identify studies. Specify the date when each source was last searched or consulted.	Pg 5
Search strategy	7	Present the full search strategies for all databases, registers, and websites, including any filters and limits used.	Pg 5
Selection process	8	Specify the methods used to decide whether a study met the inclusion criteria of the review, including how many reviewers screened each record and each report retrieved, whether they worked independently, and if applicable, details of automation tools used in the process.	Pg 6
Data collection process	9	Specify the methods used to collect data from reports, including how many reviewers collected data from each report, whether they worked independently, any processes for obtaining or confirming data from study investigators, and if applicable, details of automation tools used in the process.	Pg 6
Data items	10a	List and define all outcomes for which data were sought. Specify whether all results that were compatible with each outcome domain in each study were sought (e.g. for all measures, time points, analyses), and if not, the methods used to decide which results to collect.	Pg 7
	10b	List and define all other variables for which data were sought (e.g. participant and intervention characteristics, funding sources). Describe any assumptions made about any missing or unclear information.	
Study risk of bias assessment	11	Specify the methods used to assess risk of bias in the included studies, including details of the tool(s) used, how many reviewers assessed each study and whether they worked independently, and if applicable, details of automation tools used in the process.	Pg 7
Effect measures	12	Specify for each outcome the effect measure(s) (e.g. risk ratio, mean difference) used in the synthesis or presentation of results.	Pg 7
Synthesis methods	13a	Describe the processes used to decide which studies were eligible for each synthesis (e.g. tabulating the study intervention characteristics and comparing against the planned groups for each synthesis (item #5)).	Pg 7
	13b	Describe any methods required to prepare the data for presentation or synthesis, such as handling of missing summary statistics, or data conversions.	Pg 7
	13c	Describe any methods used to tabulate or visually display results of individual studies and syntheses.	Pg 7
	13d	Describe any methods used to synthesize results and provide a rationale for the choice(s). If meta-analysis was performed, describe the model(s), method(s) to identify the presence and extent of statistical heterogeneity, and software package(s) used.	Pg 7
	13e	Describe any methods used to explore possible causes of heterogeneity among study results (e.g. subgroup analysis, meta-regression).	Pg 7
	13f	Describe any sensitivity analyses conducted to assess robustness of the synthesized results.	
Reporting bias assessment	14	Describe any methods used to assess risk of bias due to missing results in a synthesis (arising from reporting biases).	Pg 7
Certainty assessment	15	Describe any methods used to assess certainty (or confidence) in the body of evidence for an outcome.	Not done
Results			
Study selection	16a	Describe the results of the search and selection process, from the number of records identified in the search to the number of studies included in the review, ideally using a flow diagram.	Pg 7-8
	16b	Cite studies that might appear to meet the inclusion criteria, but which were excluded, and explain why they were excluded.	Pg 7-8
Study characteristics	17	Cite each included study and present its characteristics.	Pg 10- 14
Risk of bias in studies	18	Present assessments of risk of bias for each included study.	Pg 15
Results of individual studies	19	For all outcomes, present, for each study: (a) summary statistics for each group (where appropriate) and (b) an effect estimate and its precision (e.g. confidence/credible interval), ideally using structured tables or plots.	Pg 16 and 17
Results of syntheses	20a	For each synthesis, briefly summarise the characteristics and risk of bias among contributing studies.	Pg 16 and 17
	20b	Present results of all statistical syntheses conducted. If meta-analysis was done, present for each the summary estimate and its precision (e.g. confidence/credible interval) and measures of statistical heterogeneity. If comparing groups, describe the direction of the effect.	Pg 16 and 17
	20c	Present results of all investigations of possible causes of heterogeneity among study results.	Pg 16 and 17
	20d	Present results of all sensitivity analyses conducted to assess the robustness of the synthesized results.	Pg 16 and 17
Reporting biases	21	Present assessments of risk of bias due to missing results (arising from reporting biases) for each synthesis assessed.	Pg 16 and 17
Certainty of evidence	22	Present assessments of certainty (or confidence) in the body of evidence for each outcome assessed.	Pg 16 and 17
Discussion			
Discussion	23a	Provide a general interpretation of the results in the context of other evidence.	Pg 19
	23b	Discuss any limitations of the evidence included in the review.	Pg 20
	23c	Discuss any limitations of the review processes used.	Pg 20
	23d	Discuss implications of the results for practice, policy, and future research.	Pg 20
Other information			
Registration and protocol	24a	Provide registration information for the review, including register name and registration number, or state that the review was not registered.	Not registered
	24b	Indicate where the review protocol can be accessed, or state that a protocol was not prepared.	Protocol was prepared
	24c	Describe and explain any amendments to information provided at registration or in the protocol.	None
Support	25	Describe sources of financial or non-financial support for the review, and the role of the funders or sponsors in the review.	Pg 21
Competing interests	26	Declare any competing interests of review authors.	Pg 21
Availability of data, code, and other materials	27	Report which of the following are publicly available and where they can be found: template data collection forms; data extracted from included studies; data used for all analyses; analytic code; any other materials used in the review.	With investigator
